# Polyvalent Bacterial Lysate Protects Against Pneumonia Independently of Neutrophils, IL-17A or Caspase-1 Activation

**DOI:** 10.3389/fimmu.2021.562244

**Published:** 2021-04-26

**Authors:** Florencia Ferrara, Analía Rial, Norma Suárez, José Alejandro Chabalgoity

**Affiliations:** Laboratory for Vaccine Research, Departamento de Desarrollo Biotecnológico, Facultad de Medicina, Instituto de Higiene, Montevideo, Uruguay

**Keywords:** polyvalent bacterial lysate, respiratory tract infections, TLR stimulation, pneumonia, lungs, IL-17A, inflammasome

## Abstract

Polyvalent bacterial lysates have been in use for decades for prevention and treatment of respiratory infections with reported clinical benefits. However, besides claims of broad immune activation, the mode of action is still a matter of debate. The lysates, formulated with the main bacterial species involved in respiratory infections, are commonly prepared by chemical or mechanical disruption of bacterial cells, what is believed influences the biological activity of the product. Here, we prepared two polyvalent lysates with the same composition but different method of bacterial cell disruption and evaluated their biological activity in a comparative fashion. We found that both bacterial lysates induce NF-kB activation in a MyD88 dependent manner, suggesting they work as TLR agonists. Further, we found that a single intranasal dose of any of the two lysates, is sufficient to protect against pneumococcal pneumonia, suggesting that they exert similar biological activity. We have previously shown that protection against pneumococcal pneumonia can also be induced by prior *S. pneumoniae* sub lethal infection or therapeutic treatment with a TLR5 agonist. Protection in those cases depends on neutrophil recruitment to the lungs, and can be associated with increased local expression of IL-17A. Here, we show that bacterial lysates exert protection against pneumococcal pneumonia independently of neutrophils, IL-17A or Caspase-1/11 activation, suggesting the existence of redundant mechanisms of protection. Trypsin-treated lysates afford protection to the same extent, suggesting that just small peptides suffice to exert the protective effect or that the molecules responsible for the protective effect are not proteins. Understanding the mechanism of action of bacterial lysates and deciphering the active components shall allow redesigning them with more precisely defined formulations and expanding their range of action.

## Introduction

Respiratory tract infections (RTIs) are the most frequent among all human infections, associated with high morbidity and mortality, and affecting mainly children and the elderly (1). *Streptococcus pneumoniae* is the leading cause responsible for almost 50% of cases, followed in prevalence by *Haemophilus influenzae* with an incidence of 20%. *Staphylococcus aureus* and *Klebsiella pneumoniae* are also involved, as well as several viral pathogens, such as respiratory syncytial virus and Influenza (2, 3). Although knowledge about major respiratory pathogens and their interaction with the host have increased significantly in recent years, prevention and treatment of RTIs remain a major public health challenge. There is a clear need for new strategies and therapeutic alternatives to control the burden of RTIs particularly in the lights of emergent new pathogens as the SARS-CoV-2. Given the broadness of agents involved, non-specific immunotherapies that work independently of infectious agents, strains or serotypes, seems to be central (4). During the 70s, an immunotherapy based on polyvalent bacterial lysates (PBL) formulated with the main bacterial species implicated in the RTIs emerged as a new strategy. These strains are grown independently, harvested and then lysed and mixed to obtain the PBLs used in the clinics. The cell lysis is a key stage for obtaining bacterial lysates that works effectively and different methods have been studied, being the most used alkaline and mechanical lysis (5–8). PBL can be administered orally, nasally or sublingually, and are capable of activating mucosal-associated lymphoid tissues, both locally and distally (7, 9). The epithelial cells, macrophages and dendritic cells underlying these tissues would interact directly with the bacterial lysates, mainly through TLRs, activating the NFkB and/or MAPK pathways (10–13). This activation results in the production of pro-inflammatory intermediates such as cytokines and chemokines, which then recruit effector cells and generate an acute inflammatory response in the lung (10, 13, 14). Several clinical trials have tested bacterial lysates in both adult and child populations, and encouraging results have been obtained. They were effective in preventing recurrent infections in children (15–17), as well as in adults, with a reduction in the incidence and duration of the disease (18, 19). Also in patients with Chronic Obstructive Pulmonary Diseases (COPDs), it has been shown that PBL treatment reduces the duration and severity of exacerbations (20–22). However, its use remains a matter of debate as it has been questioned the forcefulness of clinical trials and their experimental design (7). The focus has long been on the understanding of its mechanism of action in detail and looking for strategies for a better characterization of its individual components. Although in the recent years significant progress has been made, there is still a need to elucidate specific immune mechanisms involved in a common mechanism of action to such a complex mixture of antigens. This knowledge will help in the implementation of bacterial lysates as immunotherapy for the prevention and treatment of RTIs. In this work we produced polyvalent bacterial lysates by two methods, alkaline and mechanical and compared them. We found that a single dose of either bacterial lysate suffices to induce protection against lethal pneumococcus challenge, suggesting that they both have similar biological activity. Then we analyzed for one of them, whether this protection relies on the same immune mediated mechanism that have shown essential for protection against pneumococcal pneumonia exerted by other treatments. We found that intranasal administration of PBL rapidly recruits neutrophils to the lungs, but these are not necessary for the protection. Treatment and challenge experiments in *Il-17a* or *Capase-1* deficient mice also showed that protection could be fully achieved in these animals. Finally, we showed that trypsin-treated lysates afford protection to the same extent, suggesting that just small peptides suffice to exert the protective effect or that the molecules responsible for the protective effect are not proteins.

## Materials and Methods

### Growth and Culture of Bacterial Strains

*Staphylococcus aureus* (ATCC^®^ 25923 ™), *Klebsiella pneumoniae* (ATCC^®^ 10031 ™) and *Haemophilus influenzae* (ATCC^®^ 19418 ™) were grown using a vegetable medium containing vegetal Peptone Soya (40 g/L), NaCl (2 g/L), Na2HPO4 (2 g/L), Sodium (0.5 g/L) and Glucose (6 g/L). Hemin and NADH (25g/L of each) were added for *H. influenzae* culture. *Streptococcus pneumoniae* (ATCC^®^ BAA334 ™) was grown using synthetic medium as described (23). The strains were grown individually at 37°C in atmosphere with 5% CO_2_ for *H. influenzae* and *S. pneumoniae*. Overnight cultures were 1/100 diluted and incubated for 4 h or until reach an O.D. at 600 nm of 0.8-1. The biomass was harvested by centrifugation, washed and finally resuspended in saline solution. For the *in vivo* infection model with *S. pneumoniae*, a serotype 1 clinical isolate (E1586, Sp1) obtained from the National Reference Laboratory, Ministry of Health, Montevideo, Uruguay was used. Working stocks of Sp1 were prepared in Todd Hewitt broth (Sigma) supplemented with 0.5% yeast extract (THYB) and stored at −80°C in THYB plus 12% (vol/vol) glycerol for no longer than six months as previously described (24–27). The consistency of batch production of working stocks, was assessed by checking that the minimal lethal dose (MLD) is maintained, and that protection against a MLD challenge is obtained by treatment with flagellin and/or a sublethal infection as before (24, 25).

### Bacterial Lysates

Alkaline and mechanical lysates for each bacterial strain were prepared as follow. For alkaline lysis, bacterial cultures were first inactivated (100°C for 10 minutes). Then, several NaOH concentrations were assessed (at controlled temperature and pH), in a kinetic analysis were aliquots were obtained at different time-points and their bacterial profiles were analyzed by SDS-PAGE. Once optimal conditions (NaOH concentration and time of lysis) were selected for each strain, the alkaline lysis was performed and neutralized with HCl. After centrifugation, the supernatant was washed and filtered on the Quix Stand benchtop system (GE HealthCare) with a 30 kDa membrane. For mechanical cell disruption an EmulsiFlex - C3 Disruptor (Avestin, Inc.) was used. The homogenization pressure was set between 500 and 30000 psi. The protein profiles of the different fractions obtained during the passages were analyzed by SDS-PAGE. Once the optimal number of passages was selected, the bacterial biomass was passed through, the lysate was collected, centrifuged and the supernatant preserved. Proteins were quantified by the BCA method. Protein profiles were determined by SDS-PAGE on 12% gels. Carbohydrate quantification was performed by the Phenol - Sulfuric Acid method, using a standard glucose curve as previously reported (28). The polyvalent alkaline bacterial lysate (PABL) and polyvalent mechanical bacterial lysate (PMBL) were formulated by mixing equal amounts (250 μg of total proteins) of each monovalent bacterial lysates obtained by alkaline or mechanical lysis. For *in vivo* or *in vitro* assays, the same total protein amount of PABL or PMBL were used. For trypsinization of the bacterial lysates, two volumes of PABL or PMBL were mixed with 1 volume of 0.017% Trypsin-EDTA and incubated at 37°C with shaking for 1 h. The mixture was then incubated at 70°C for 30 minutes. Complete trypsinization was confirmed by SDS-PAGE analysis observing absence of proteins.

### *In Vitro* Stimulation of NF-kB and/or AP-1 Reporter Cells

The production of SEAP reporter gene (secreted embryonic alkaline phosphatase) was evaluated in culture supernatants taken 24 h after stimulation with the lysates (at 37°C with 5% CO2), using the QUANTI-Blue method (Invivogen). THP1-XBlue cells (Invivogen) were cultured in RPMI 1640 medium (PAA) supplemented with 2 mM L-glutamine, 4.5 g/L glucose, 10 mM HEPES, 1 mM sodium pyruvate, 10% (v/v) of inactivated FBS (Capricorn), 100 μg/ml Normocin (Invivogen), Penicillin - Streptomycin (50 U/ml - 50 μg/ml) and 200 μg/ml Zeocin (Invivogen) as selective antibiotic. Cells were seeded in 96-well plates (2x10^5^ cells/well) and stimulated for 24 h with 10 μg/ml of PABL or PMBL. THP1-XBlue-defMyD cells (Invivogen) were cultured in the same medium as THP1-XBlue cells with the addition of the selection antibiotic Hygromycin B Gold (100 μg/ml, Invivogen). RAW-Blue cells (Invivogen) were cultured in DMEM with glucose (4.5 g/L, PAA) supplemented with 2 mM L-glutamine, 10% (v/v) FBS, 100 μg/ml Normocin and 200 μg/ml Zeocin. Cells, suspended in analysis medium containing inactivated FBS instead of FBS, were seeded in 96-well plates (1 x 10^5^ cells/well) and stimulated with 10 μg/ml of PMBL or PMBL previously tripsinized.

### *In Vitro* Stimulation of Alveolar Epithelial Cells

A549 cells (ATTC CCL-185) were grown on Ham’s F-12 medium (Capricorn) supplemented with 5% v/v of FBS. Cells were plated in 24-well plates (2x10^5^ cells/well) and 24 h later were stimulated with 10 μg/ml of PABL or PMBL, for several times ranging from 1 to 48 h. At selected time-points, the medium was removed, cells were washed with phosphate-buffered saline (PBS) and lysed in TRIzol (Invitrogen) for total RNA extraction as described below.

### Animal Studies

Female mice of C57BL/6, *Il17a-/-*, *Casp1-/-* and *Casp1+/+* strains (6 to 8 week-old) supplied by the National Division of Veterinary Laboratories or the Transgenic and Experimental Animals Unit of the Pasteur Institute of Montevideo (Uruguay) were used for the experiments. Animals were maintained in individually ventilated cages and were handled in a vertical laminar flow cabinet (Class II A2; Esco, Hatboro, PA). All experiments complied with current national and institutional regulations and ethical guidelines (CHEA, Uruguay). Mice were anesthetized by intraperitoneal (i.p.) injection of 2.2 mg ketamine plus 0.11 mg xylazine in a total volume of 200 µl. PABL or PMBL were diluted in sterile saline solution and administered into the nostrils of mice, at the desired dose (6 or 18 μg) in a final volume of 30 μl. For mouse infection protocols, working stocks of Sp1 were thawed, washed with sterile saline solution and diluted to the appropriate dose. One MLD of Sp1 was administered intranasally in a final volume of 50 μl to previously anesthetized mice, and in each experiment the bacterial inoculum was confirmed after infection by plating serial dilutions onto blood agar plates. Mice survival was followed up for at least 2 weeks after Sp1 challenge

For depletion of granulocytes, 100 µg of anti-Gr-1 (RB6-8C5) or an isotype control (HB152) was administered i.p. 12 h before i.n. administration of PMBL. Sp1 challenge was performed 24 h after PMBL administration. The anti-Gr1 injection was found to completely deplete PMNs in peripheral blood as assessed by flow cytometry, as reported before (25). We confirmed that the administration of RB6-8C5 induce a 99% reduction in PMNs assessed in peripheral blood circulation 24 h after PMBL treatment, compared with those receiving the isotype control antibody (**Supplemental Figure 1**).

### Flow Cytometry

For bronchoalveolar lavages (BAL) sampling, the trachea was cannulated, and 1 ml of PBS plus 1 mM EDTA was instilled six times and recovered by gentle aspiration. BAL cells were washed in PBS containing 2 mM EDTA and 1% fetal bovine serum (FACS-EDTA). For immunophenotyping, 1x10^6^ cells were seeded per tube, and Fc receptors were blocked by incubation with 1 μl of Fc Block (BD) for 20 minutes at 4°C. Then, cells were labeled with specific monoclonal antibodies (BD) (Ly6G-PE, CD11b-APC-Cy7) for 30 minutes at 4°C and finally washed and fixed with 4% formaldehyde and stored at 4°C in dark. Cells were acquired into a FACS Canto II cytometer using BD FACSDiva™software (BD) for acquisition and analysis.

### qRT-PCR

Total RNA extraction was performed from cultured cells or lung portions, preserved in TRIzol (Invitrogen), following the manufacturer’s instructions. Lungs were homogenized with a TissueRuptor (Qiagen). Quantification of nucleic acids was performed in Nanodrop (Thermo Fisher Scientific). 1 μg total RNA was treated with DNase I (Invitrogen), and first-strand cDNA synthesis was carried out using random primers (Invitrogen) and Moloney murine leukemia virus (M-MLV) reverse transcriptase (Invitrogen) in a Corbett CG1-96 thermocycler (10 min at 25°C, 50 min at 37°C, 15 min at 70°C). qPCR was performed using specific primers at a final concentration of 1 μM and a QuantiTect SYBR green PCR kit (Qiagen) in a Applied Biosystem 7900 HT Fast Real Time PCR (15 min at 95°C, followed by 40 cycles at 95°C for 15 sec and 60°C for 1 min). Differences in gene expression level were obtained using the Ct method for relative mRNA quantitation using β-actin or β2-microglobulin as housekeeping genes. To ensure the reliability of qRT-PCR results, we first evaluated three different housekeeping genes to check that they do not change between different experimental conditions, and based on the results, we choose the single best for all further experiments. In the experiments presented here we tested GAPDH, β-actin and β2-microglobulin for A549 human cell line, and GAPDH, 18S and β-actin for experiments in mice, and then selected β2-microglobulin for the former and β-actin for the later. Further, for every experiment conducted, results were first validated by double-checking that expression of the selected housekeeping gene showed no variation between samples, i.e. Ct values for the housekeeping gene among samples has to show less than 1 cycle of variation.

### Statistical Analysis

GraphPadPrism7 Software (GraphPad Software, San Diego, CA) was used. The one-way ANOVA test was used to compare means between groups and two-way ANOVA, with Bonferroni correction, for comparison of means between groups and at different times. A log rank (Mantel-Cox) test was performed for analysis of Kaplan-Meier survival curves.

## Results

### Preparation and Characterization of Polyvalent Bacterial Lysates

It has been argued that the means by which the lysates are prepared (mechanical vs chemical disruption of bacterial cells) has direct influence in the immunostimulant activity of the product (5). Thus, we prepared two different PBL with the same bacterial composition but using either mechanical (PMBL) or alkaline treatment (PABL) as method of bacterial disruption, for side-by-side comparison. Higher number of proteins and carbohydrates were obtained in the mechanical version of the lysates, in monovalent as well as in the polyvalent lysates (**Table 1**). In addition, SDS-PAGE analysis of individual bacterial lysates, showed that those prepared by mechanical disruption show larger number and more defined protein bands, whereas alkaline lysates showed a smear in the SDS-PAGE (**Supplemental Figure 2**). In line with this, we have also recently showed elsewhere that MALDI-TOF spectra revealed more proteins abundance and with greater intensity in the PMBL compared to PABL (29). For use in comparative analysis PABL were further concentrated as to have the same amount of total proteins than PMBL.

**Table 1:** Proteins and carbohydrates contents of individual lysates obtained by alkaline or mechanical lysis as described in *Methods*.

	Proteins (mg/ml)		Carbohydrates (mg/ml)	
	Alkaline PABL	Mechanical PMBL	Alkaline PABL	Mechanical PMBL
***Staphylococcus aureus***	0.21 ± 0.01	0.51 ± 0.02	0.02 ± 0.01	0.49 ± 0.06
***Klebsiella pneumoniae***	1.02 ± 0.01	1.29 ± 0.02	0.30 ± 0.04	0.40 ± 0.01
***Streptococcus pneumoniae***	0.18 ± 0.01	0.41 ± 0.01	0.71 ± 0.09	0.70 ± 0.05
***Haemophilus influenzae***	0.24 ± 0.01	0.49 ± 0.01	0.08 ± 0.04	0.18 ± 0.09
**Polyvalent lysate**	0.22 ± 0.01	0.59 ± 0.01	0.20 ± 0.01	0.46 ± 0.01

The data represent the mean of n=3 replicate ± standard deviation (S.D.) and are representative of at least 3 distinct batches of each culture.

### Polyvalent Bacterial Lysates Activates NF-kB in a MyD88 Dependent Manner

**Figure 1:**
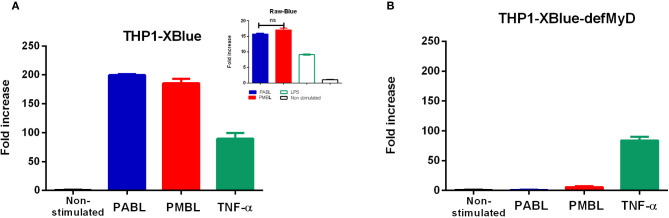
THP1-Xblue **(A)**, RAW-Blue **(A**, inset) and THP1-XBlue-DefMyD **(B)** cells were in vitro stimulated for 24 h with PABL or PMBL (10 mg/ml). TNF-α (50 ng/ml) was used as a control of activation in MyD88-deficient cells. LPS (1μg/ml) was used as a control of activation of RAW-Blue cells. SEAP reporter was quantified in culture supernatants by optical density readings at 638 nm using the QUANTI-Blue method. The bars represents Mean ± SEM of triplicate cultures for relative increments of stimulated cells related to non-stimulated ones. Data are representative of three independent experiments. ns, non-significant difference.

*In vitro* stimulation of the THP-1 XBlue reporter cell line with either PMBL or PABL induced similar increase of NF--kB/AP-1 activity (**Figure 1A**). Instead, neither lysate induced production of the reporter protein when used to stimulate the MyD88-deficient THP-1 cell line, confirming that the activation of NF-kB and/or AP-1 induced by the polyvalent bacterial lysates is MyD88-dependent (**Figure 1B**). A similar result was obtained when a murine reporter cell line (RAW-Blue) was used (**Figure 1A** inset).

### Mechanical Lysate Induces Stronger Pro-Inflammatory Activity in Human Alveolar Cells

**Figure 2:**
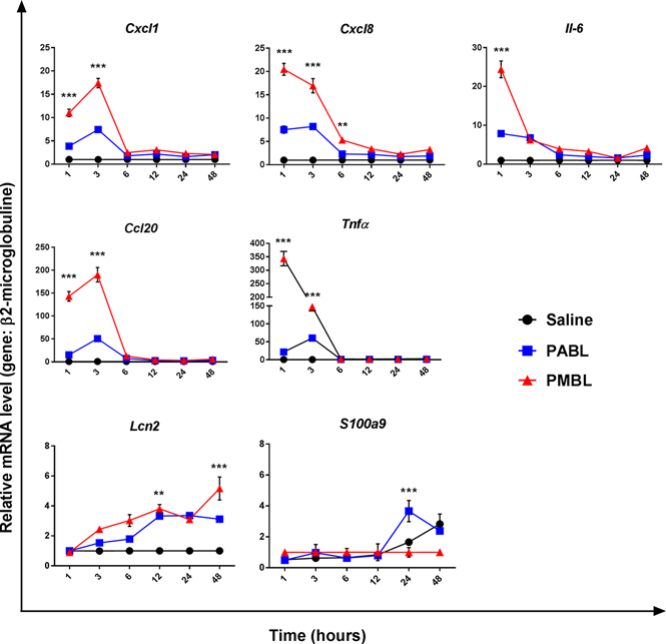
Temporally kinetics of the transcriptional profile of the alveolar cell line (A549) *in vitro* stimulated with 10 μg/ml of either PABL or PMBL for 1, 3, 6, 12, 24 or 48 h. Relative mRNA levels of *Ccl20, Cxcl1, Cxcl8, Tnfa, Il6* and *Lipocalin 2* were assessed by RT-qPCRs. Data are expressed as fold increase of relative mRNA levels for each gene normalized against β*2-microglobulin* referred to non-stimulated cultures. Each point represents Mean ± SEM of quintuplicate cultures. Data are representative of three independent experiments. Significant differences between groups are indicated by **p < 0.01 and ***p < 0.001, two-way ANOVA with Bonferroni correction.

We have previously shown that the human alveolar epithelial cell line A549 stimulated with an alkaline PBL engages in a pro-inflammatory gene expression program within 4 h after stimulation (26). Thus, we stimulated side-by-side A549 cells with either polyvalent bacterial lysate, and followed up the magnitude and kinetics of that transcriptional profile for up to 48 hours. We found that both lysates rapidly upraised the transient gene expression of several pro-inflammatory chemokines (*Ccl20, Cxcl8, Cxcl1*) and cytokines (*Il6, Tnfα*) as well as of antimicrobial peptides (*Lcn2, S100a9*). However, in most cases PMBL induced significantly higher mRNA levels than PABL (**Figure 2**). Upregulation of chemokines and cytokines peaked by 1 or 3 h after stimulation, and returned to basal level by 6 h after stimulation. Instead, Lipocaline 2 (*Lcn2*) and *S100a9* showed a different kinetics. Whereas expression level of *Lcn2* was already increased by 3 hours and kept on rising by 48 h, differential expression of *S100a9* was only apparent after 24 h of stimulation (**Figure 2**). *DEFB1 (*human beta defensin 1) and *Camp* (Cathelicidine antimicrobial peptide) were also assessed but no significant increases in mRNA levels were detected at any time-point (**Supplemental Figure 3**).

### Both Polyvalent Bacterial Lysates Induces Similar Pro-Inflammatory Activity in Lungs

**Figure 3:**
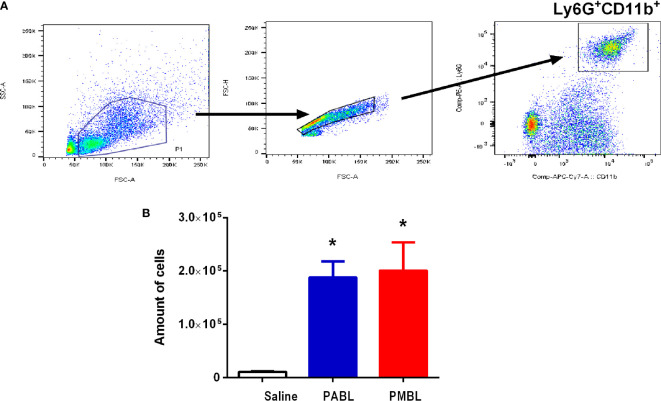
Neutrophils from the bronchoalveolar lavages (BAL) were obtained from mice pre- treated with the polyvalent lysates. Mice (n=6) were given i.n. PABL, PMBL (6 μg/30 μl) or saline and sacrificed 24 h later. **(A)** Gating strategy; neutrophils were defined by forward scatter area (FSC-A) and side scatter area (SSC-A) profile, doublet discrimination, and high expression of CD11b+ and Ly6G+. **(B)** Bars represent Mean ± SEM of BAL neutrophils for each group. Significant differences are indicated by *p < 0.05, treated vs. untreated group, one-way ANOVA. Data are representative of three independent experiments.

**Figure 4:**
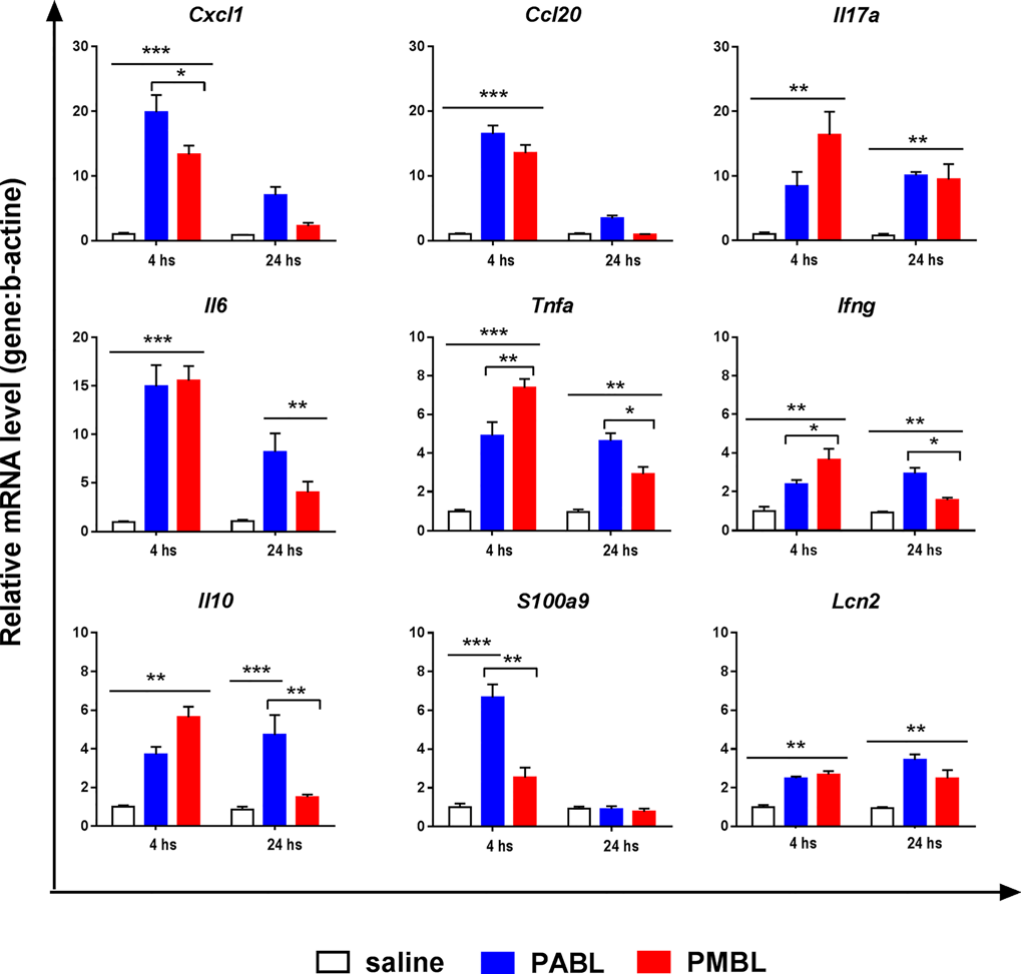
Pro-inflammatory response induced in the lungs of mice treated with either PABL or PMBL. Mice received i.n. PABL or PMBL (6 µg/30 µl) or saline, and lungs were obtained 4 or 24 hours later. Relative mRNA levels for *Cxcl1, Ccl20, Il17a, Il6, Tnfa, Infγ, Il10, S100a9* and *Lipocalin 2* were assessed by RT-qPCRs. Data is expressed as fold increase of relative mRNA levels of each gene normalized against b-actin referred to non-stimulated cultures. Each point represents Mean ± SEM of quintuplicate cultures. Significant differences between groups are indicated by *p < 0.05, **p < 0.005 and ***p < 0.0001, one-way ANOVA. Data are representative of three independent experiments.

Mice received intranasally any of each polyvalent lysate and 24 h later were sacrificed to evaluate the inflammatory response induced *in vivo*. Both lysates induced a significant influx of neutrophils into the lungs, showing an approximate 20-fold increment as compared with the saline-treated group (**Figure 3**). No difference in PMN recruitment was observed between PABL- or PMBL-treated groups. Further, a rapid inflammatory gene expression program was induced in both treated groups (**Figure 4**). Expression of pro-inflammatory mediators was up regulated by 4 h after treatment, and whereas for some of them expression levels decreased at 24 h (*Il6, Cxcl1*, *Ccl20*, *S100a9*), others maintained their high expression level (*Il10*, *Tnf-α*, *Il17a*, *Ifn-γ*, *Lcn2*). Both polyvalent lysates generated a similar pro inflammatory response, yet with differences at particular time points.

### Polyvalent Bacterial Lysates Protects Against Pneumococcal Pneumonia

**Figure 5:**
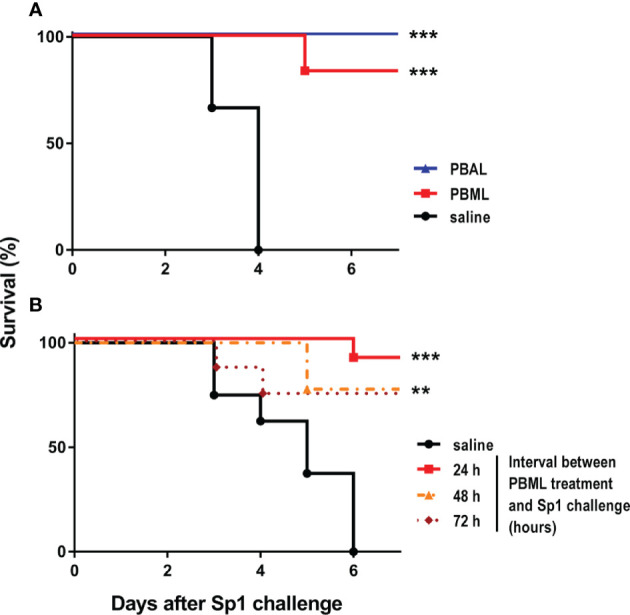
Survival after *Streptococcus pneumoniae* (Sp1) challenge of mice pretreated with the Polyvalent Bacterial lysates. **(A)** Mice (n=6) received i.n. PABL or PMBL (18µg/30 µl) and 24 h later were challenged i.n. with the MLD of Sp1 (3x10^6^ CFU/50 µl). **(B)** Mice (n=6) were i.n. treated with PMBL (18µg/30 µl) at 72, 48 and 24 h before being challenged with the MLD of Sp1 (3x10^6^ CFU/50 µl). Survival rates were recorded for at least 7 days after challenge. Significant differences are shown by **p < 0.001, ***p < 0.0001, treated vs. untreated, Log-rank test (Mantel-Cox). Data are representative of more than three independent experiments.

Mice were treated with one dose of either PABL or PMBL, and 24 h later they were challenged with a lethal dose of *S. pneumoniae* serotype 1 (Sp1). Treatment with either lysate conferred protection against pneumococcal lethal pneumonia with survival rates of 80 and 100% for PMBL and PABL, respectively (**Figure 5A**). We then evaluated whether PMBL treatment afforded protection when the challenge was administered later in time. Lysates administered either 48 or 72 h before pneumococcal challenged still afforded significant protection (**Figure 5B**).

### Protection Does Not Depend on Neutrophil Recruitment to the Lungs

**Figure 6:**
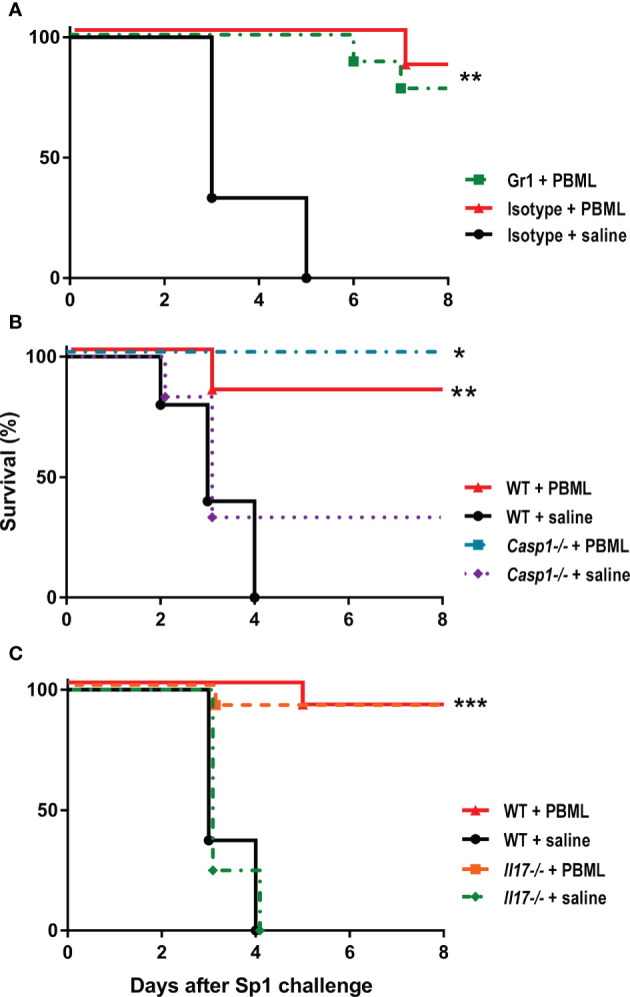
**(A)** Survival rates after *Streptococcus pneumoniae* (Sp1) challenge of mice that were depleted from PMNs. Mice received Gr-1 or isotype control antibodies (i.p.) 12 h before being treated i.n. with PMBL lysate. All mice were challenged 24 h later with the MLD of Sp1 (3x10^6^ CFU/50 µl). **(B)** Survival rates of *Casp1* and wild-type mice (littermate of *Casp1*) n=6, challenged with Sp1 24 h after treatment with PMBL (18µg/30 µl) or saline. **(C)** *Il17a-/-* and wild-type mice n=6 were challenged i.n. with Sp1 24 h after treatment with PMBL (18µg/30 µl) or saline. Survival rates were recorded for 8 days for each group (n=6). Significant differences are shown by *p < 0.01, **p < 0.001, ***p < 0.0001 treated vs. untreated, Log-rank test (Mantel-Cox). Data are representative of three independent experiments.

We have previously reported that intranasal administration of a sublethal dose of pneumococcus, or intranasal stimulation with flagellin, both induces high level of resistance to *S. pneumoniae* (Sp1) lethal challenge (24, 25). Protection depended on neutrophils in both cases. Thus, we tested whether neutrophils are also required for PMBL-mediated protection. For that, 12 h before PMBL treatment, one group of mice received anti-Gr-1 antibody that depletes up to 99% of the neutrophil population and another group received an isotype control antibody (**Supplemental Figure 1**). Both groups of animals receiving PMBL lysate survived a further Sp1 challenge whether or not neutrophils were depleted (**Figure 6A**). Thus, protection from lethal pneumonia induced by the PMBL lysate does not depend on neutrophils.

### Protection Does Not Require IL-17A nor Caspase-1 Activation

IL-17A has been reported as relevant cytokine for protection against *S. pneumoniae* (30). We have previously shown that IL-17 expression was strongly up regulated in sub lethal infected animals that were protected against *S. pneumoniae* challenge (24, 26), and here we found that *Il17a* is also upraised after bacterial lysates treatment (**Figure 4**). Thus, we evaluated whether is required for PMBL-mediated protection. *Il17a^-/-^* and WT mice were treated with PMBL and then challenged with Sp1. Again, both groups showed same survival rates, suggesting that *Il17a* is not required for the resistance against pneumonia induced by PMBL (**Figure 6C**). To further investigate the mechanisms associated with PMBL-mediated protection against lethal pneumococcal pneumonia, we also evaluated the role of inflammasome activation. *Casp1^-/-^* or syngeneic WT mice were treated with PMBL and then challenged with Sp1. Both groups showed same survival rates (**Figure 6B**).

### Whole Protein Fraction Is Not Necessary for Induction of Protection by Polyvalent Lysates

**Figure 7:**
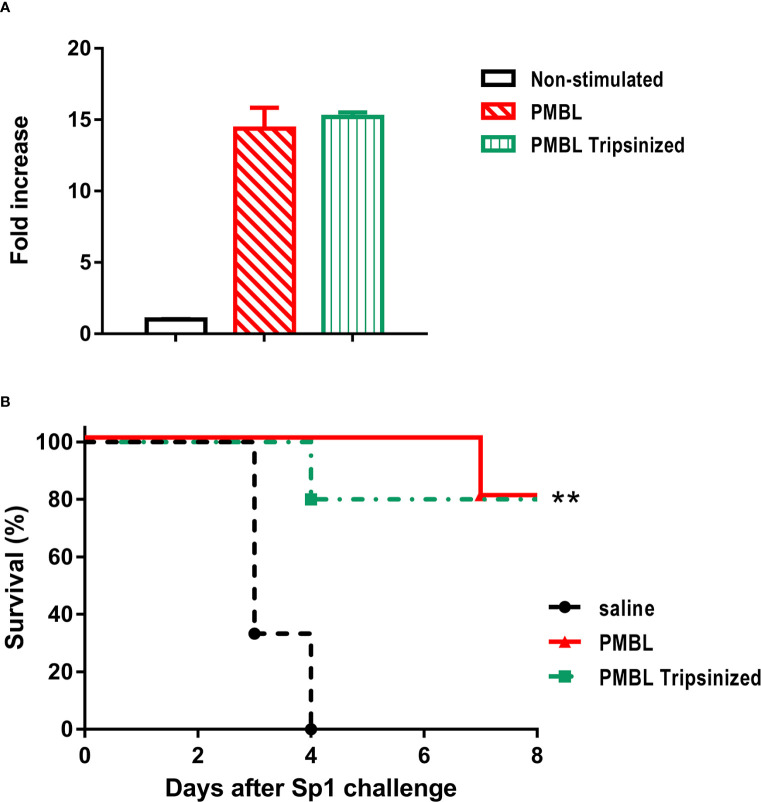
**(A)** *In vitro* stimulation of Raw-Blue cells with equivalent amounts of PMBL (10 μg/ml) or trypsinized PMBL. Supernatants were taken 24 h later and SEAP reporter levels were measured by the QUANTI-Blue method (O.D._638_). The bars represent Mean ± SEM of triplicate cultures for fold increase related to non-stimulated.Data are representative of three independent experiments. **(B)** Survival rates from lethal pneumonia challenge (Sp1) of mice (n=6) that were pretreated i.n. with same quantities (18 µg/30 µl) of PMBL and Trypsinized PMBL. All mice were challenged 24 hours later with the MLD of Sp1 (3x10^6^ CFU/50 µl). Survival rates for each group were recorded for 8 days. Significant differences are shown by **p < 0.001, treated vs. untreated, Log-rank test (Mantel-Cox). Data are representative of three independent experiments.

To evaluate the contribution of different bacteria’s component in the immunostimulant activity of PBL, we evaluated the biological activity of a trypsin-hydrolyzed PMBL lysate (PMBL tripsinized). First we assessed NF-kB/AP-1 activation, after stimulation of RAW-Blue™ reporter cells. Both, intact and hydrolyzed PMBL lysate elicited a similar response (**Figure 7A**). Then, we compared the ability to induce protection of both, intact and hydrolyzed PMBL lysate. Treatment with trypsinized PMBL or intact PMBL conferred same level of protection against pneumococcal lethal pneumonia (**Figure 7B**), confirming that protection induced by the lysates does not requires whole proteins to be present in the formulation.

## Discussion

In this work, we investigated the immunostimulant activity of PBLs, in relation with the method of preparation and their capacity to protect against pneumococcal pneumonia from a mechanistic point of view. It is postulated that the method used for bacterial cell disruption influences the biological activity of the lysate (6, 31). However, we found that two lysates prepared with the same bacterial composition and just different method of cell disruption induced MyD88-dependent NF-kB activation to a similar extent, and both protected against a lethal challenge with *S. pneumoniae*. It has been postulated that lysate-induced resistance is at least partially dependent upon TLR signaling, and the activity is lost in the absence of MyD88 (32), and this would be in line with both preparations showing the same protective activity. The only differences observed were that the mechanical lysate induced higher levels of pro-inflammatory mediators in an alveolar cell line, and that the alkaline lysate required a further step of concentration during preparation to reach the same protein concentration than the mechanical. It has been suggested that PBLs prepared by mechanical disruption of the bacterial cell are more immunogenic than those prepared by chemical lysis of the cells (31, 33). Still, here we showed that when both preparations have similar composition, they exerted similar biological effects. However, it is noteworthy that the carbohydrates to protein ratio was higher in PABL than in PMBL (**Table 1**), so when we concentrated it to equal protein concentrations then the carbohydrates content would be higher in PABL, and this could be on the basis of the improved efficacy. Altogether, these results point out the importance of better characterization of the products. Improved methods for characterization of bacterial lysate components would result in more reliable and comparable products. Most descriptions of commercial lysates, either in the literature or in patent filling just quantifies proteins and carbohydrates (5, 34–36). We have recently proposed that the use of MALDI-TOF technique for characterization and identification of biomarkers of biological activity that helps to evaluate the consistency and reproducibility of batch production would represent an advance in this sense (29).

Vaccine-induced protection against pneumonia relies on neutrophils that kill anti-capsular antibodies-opsonized bacteria (37–39). Using two other different models of protection against lethal pneumococcal challenge, we previously showed that protection is also dependent on recruitment of neutrophils to the lungs, even in the absence of antibodies (24, 25). Here, we found that bacterial lysates upregulate the expression of the neutrophil-recruiting chemokines *Cxcl-1* and IL-8 in the human alveolar epithelial cell line A549, as well the mouse functional homologue Cxcl-1 that resulted in the rapid recruitment of neutrophils into the lungs. However, neutrophils were not required for PBL-induced protection against *S. pneumoniae* challenge suggesting redundancy among protective mechanisms, since other effectors are certainly involved. The lung epithelial cells have been proposed as a key actor in antimicrobial defenses, through secretion of antimicrobial peptides. Using an aerosolized lysate of nontypeable *Haemophilus influenzae* (NTHi) it has been reported protection against *S. pneumoniae* that correlated in magnitude and time with rapid bacterial killing associated with overexpression of multiple antimicrobial polypeptides in the lung (40, 41). We found that PBLs induced the rapid expression of *Lcn2* and *S100a9* in the lungs, making tempting to speculate that they are involved in the lysate-induced protection. However, contradictory results have been reported regarding the involvement of antimicrobial peptides in lung protection. Whereas it has been shown that Lcn2 protects against *Klebsiella pneumoniae* lung infection in mice (42) and a protective role for Lcn2 in allergic airway disease has been demonstrated (43), other report has presented results showing pulmonary Lcn2 impaired bacterial clearance survival in pneumococcal pneumonia (44). Further, it has been recently reported that bronchial epithelial cells can show innate immune memory and that initial exposure to PAMPs could modify their subsequent response to infection, a phenomenon relied on epigenetic regulation (45). Thus, it is tempting to speculate that this phenomenon might be on the basis of the effectivity of the lysates.

It has been postulated that the high frequency of invasive disease associated with serotype 1 pneumococcus is because it evades sensing by inflammasomes (46). Others had shown that mucosal delivery of cholera toxin subunit B (CTB) reduces the pneumococcal load in the nasopharynx in a caspase-1/11 dependent manner (47). Instead, we found that the absence of Caspase-1 did not abrogate the protection against *S. pneumoniae*, suggesting that inflammasome sensing is not essential, and reinforcing the concept of redundancy in immune protection of the airways.

We found that the trypsinized lysates, still activates NF-kB and/or AP-1 and also protects against pneumococcal challenge. Thus, it could be that ligands such as peptidoglycans, lipopeptides, lipoteicoic acid, or even fragments of bacterial DNA or RNA are responsible of immune activation and protective effect of PBL at least against pneumococcal pneumonia. In this regard, it has been shown that aerosolized delivery of a combination of synthetic ligands for TLR2/6 and TLR9 can induce protection against both gram-negative and gram-positive pathogens as well as against influenza virus (32, 48). Beta-glucan from bacterial origin can also protect against systemic infection with *S. pneumoniae* (49). Alternatively, it could be that small peptides resulting from trypsin digestion can still be responsible of the PBL-induced protection. We previously showed that intranasal administration of the TLR5 agonist flagellin protein induces strong protection against pneumococcal mucosal challenge, but in that case the effect was lost in a trypsin-treated flagellin (25).

All in all, our results show that PBLs exert protection against *S. pneumoniae* using mechanisms distinctive from conjugate vaccine-induced protection, suggesting that these immunotherapies can be a valuable cost-effective complement for control of the burden of pneumococcal diseases. Further characterization of the active components, and their interaction with pulmonary microbiota will pave the way for the designing of new products that might be a cost-effective tool for the prevention and treatment of RTIs.

## Data Availability Statement

The original contributions presented in the study are included in the article/**Supplementary Material**. Further inquiries can be directed to the corresponding author.

## Ethics Statement

The animal study was reviewed and approved by CHEA, Comisión Honoraria de Experimentación Animal.

## Author Contributions

FF, AR, and JC contributed conception and design of the study. FF, AR, and NS carried out the experiments. FF wrote the first draft of the manuscript. JC supervised the project and wrote the manuscript. All authors contributed to the article and approved the submitted version.

## Funding

This work was funded by the National Agency of Research and Innovation (ANII, Uruguay).

## Conflict of Interest

The authors declare that the research was conducted in the absence of any commercial or financial relationships that could be construed as a potential conflict of interest.
