# Supplementary material for: Polyvalent Bacterial Lysate Protects Against Pneumonia Independently of Neutrophils, IL-17A or Caspase-1 Activation
Source: Front Immunol. 2021 Apr 26;12:562244. doi: 10.3389/fimmu.2021.562244 (PMC8108696; doi:10.3389/fimmu.2021.562244)
Supplement: Supplementary file 1 [file Image_1.pdf]

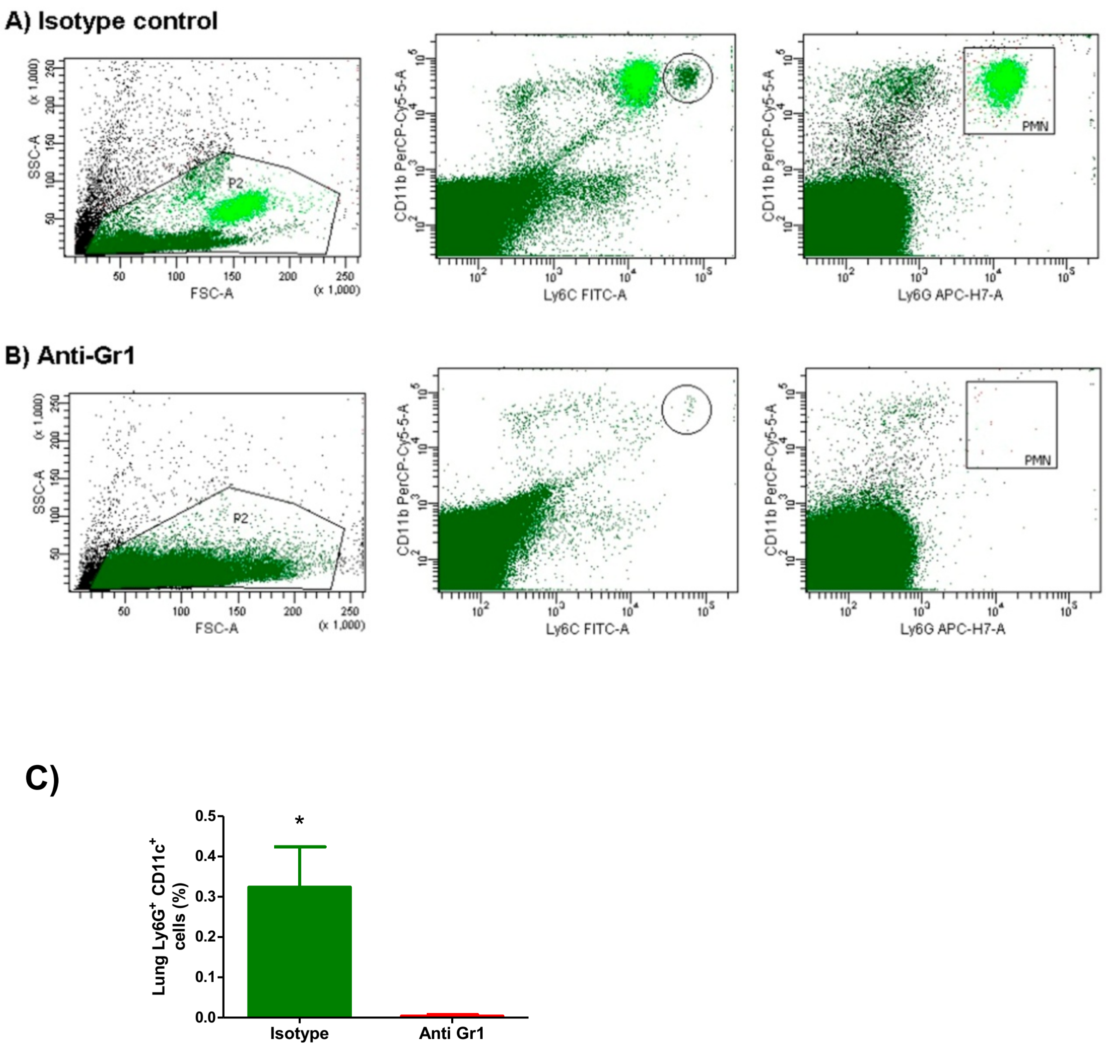

Supplemental Figure 1. Depletion of $Gr1^+$ cells. Mice (n=3) were i.p. treated with 100 µg of isotype control antibody rat IgG2b, clone HB152 (A) or monoclonal anti-Gr1 antibody clone RB6-8C5, (B) and after 12 h were i.n. treated with PMBL (18 µg/30 µl). Peripheral blood was drawn (24 h after PMBL treatment) to assess the efficacy of PMNs depletion by flow cytometry using the following antibodies CD11b-PerCPCy5, Ly6C-FITC y Ly6G-APCCy7. The administration of RB6-8C5 antibodies induced almost absolute reduction in peripheral blood PMNs populations assessed both as $CD11b^+$/$Ly6C^+$ (circle) and $CD11b^+$/$Ly6G^+$ (square) 24 h after PMBL treatment, compared with those receiving the isotype control antibody. (C) Mean ± SEM of lung $Ly6G^+$ $CD11b^+$ cells (shown as % from total lung viable cells).*, $p<0.05$, Unpaired Man Whitney Test.
