# Supplementary material for: Polyvalent Bacterial Lysate Protects Against Pneumonia Independently of Neutrophils, IL-17A or Caspase-1 Activation
Source: Front Immunol. 2021 Apr 26;12:562244. doi: 10.3389/fimmu.2021.562244 (PMC8108696; doi:10.3389/fimmu.2021.562244)
Supplement: Supplementary file 2 [file Image_2.pdf]

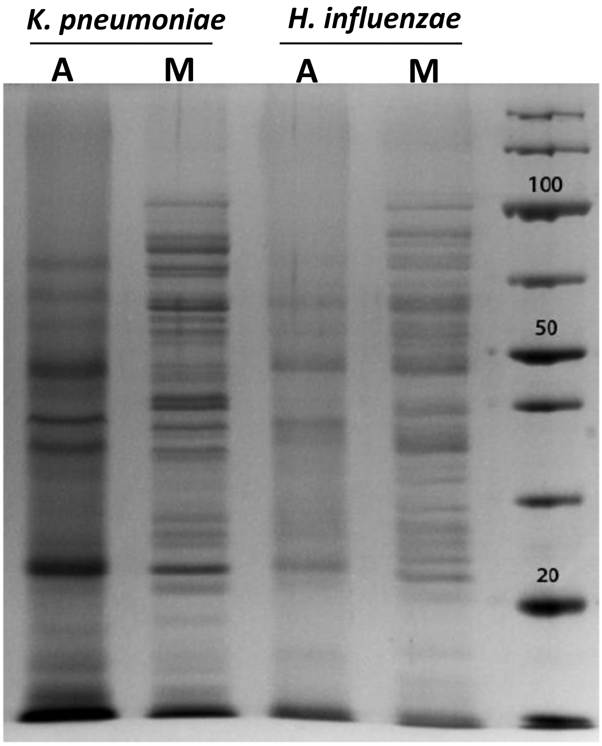

Supplemental Figure 2. SDS-PAGE protein profile for monovalent alkaline (A) and mechanical (M) lysate of *Klebsiella pneumoniae* and *Haemophilus influen*zae. 5 µg of total protein of each lysate were loaded.
