# Supplementary figures and images for: Polyvalent Bacterial Lysate Protects Against Pneumonia Independently of Neutrophils, IL-17A or Caspase-1 Activation
Source: Front Immunol. 2021 Apr 26;12:562244. doi: 10.3389/fimmu.2021.562244 (PMC8108696; doi:10.3389/fimmu.2021.562244)

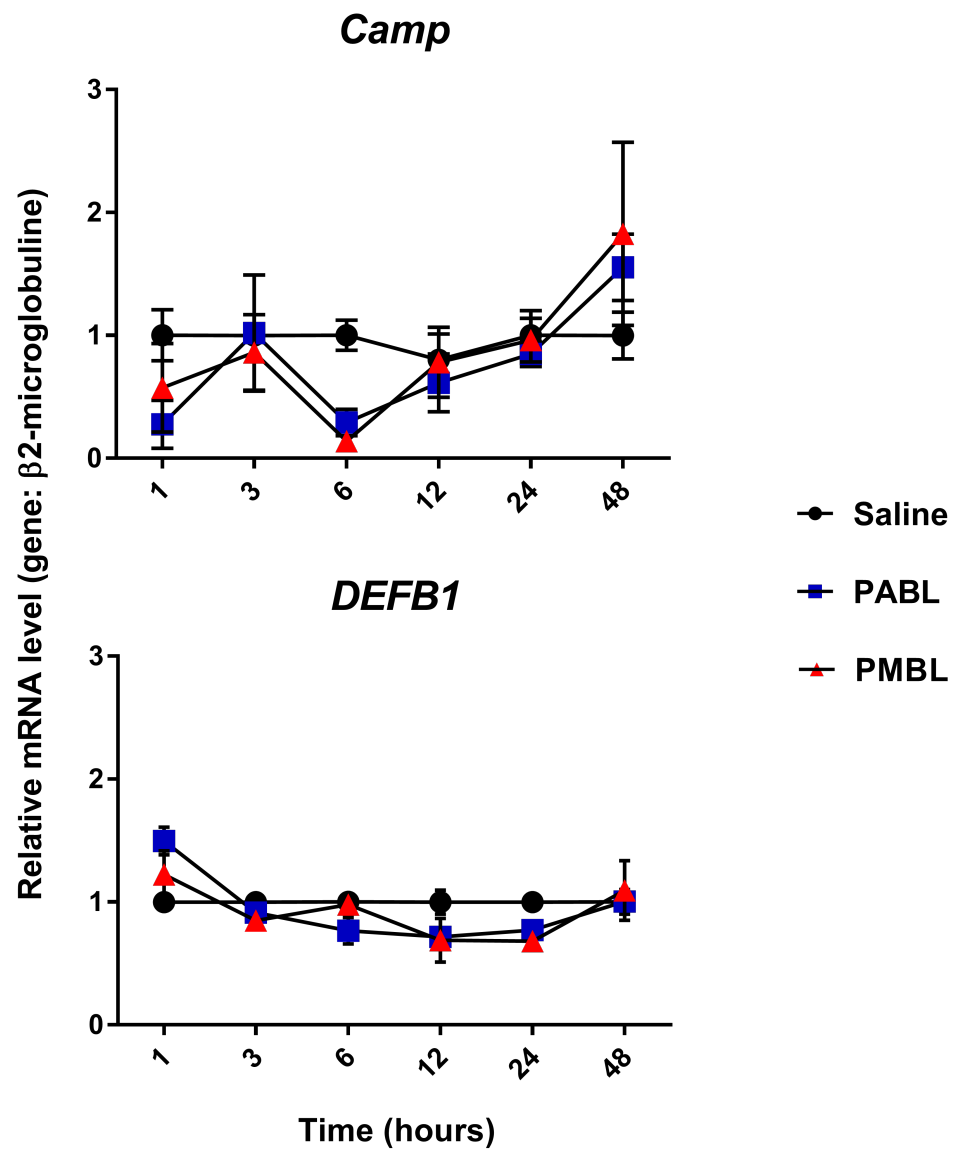

**Supplemental Figure 3.** Relative mRNA levels of *Camp*, and *DEFB1* as assessed by RT-qPCRs

Supplement: Supplementary file 3 [file Image_3.pdf]
